# Autonomic response to early head‐up tilt in patients with severe traumatic brain injury: Analysis from a randomized feasibility trial

**DOI:** 10.14814/phy2.15666

**Published:** 2023-04-20

**Authors:** Michala Dalsgaard Schultz, Morten Alstrup, Markus Harboe Olsen, Ronan M. G. Berg, Jesper Mehlsen, Kirsten Møller, Christian Gunge Riberholt

**Affiliations:** ^1^ Department of Neuroanaesthesiology, Neuroscience Centre Copenhagen University Hospital‐Rigshospitalet Copenhagen Denmark; ^2^ Department of Neurorehabilitation, Traumatic Brain Injury Copenhagen University Hospital‐Rigshospitalet Copenhagen Denmark; ^3^ Department of Clinical Physiology and Nuclear Medicine Copenhagen University Hospital‐Rigshospitalet Copenhagen Denmark; ^4^ Centre for Physical Activity Research Copenhagen University Hospital‐Rigshospitalet Copenhagen Denmark; ^5^ Department of Biomedical Sciences, Faculty of Health and Medical Sciences University of Copenhagen Copenhagen Denmark; ^6^ Neurovascular Research Laboratory, Faculty of Life Sciences and Education University of South Wales Pontypridd UK; ^7^ Section for Surgical Pathophysiology, Juliane Marie Centre Copenhagen University Hospital‐Rigshospitalet Copenhagen Denmark; ^8^ Department of Clinical Medicine, Faculty of Health and Medical Sciences University of Copenhagen Copenhagen Denmark

**Keywords:** autonomic dysfunction, heart rate variability, mobilization, orthostatic intolerance, traumatic brain injury

## Abstract

Patients with severe traumatic brain injury (TBI) may have autonomic dysfunction, one manifestation of which is orthostatic intolerance. This potentially impairs physical rehabilitation. However, the exact mechanisms remain elusive. In 30 patients participating in a trial of early tilt training versus standard care and 15 healthy volunteers, 5‐min electrocardiography was recorded in the supine position and during 70° head‐up tilt. Heart rate variability was analyzed by the low‐ and high‐frequency (LF and HF) power, the LF–HF ratio, the total power, the ratio of the standard deviation of normal‐to‐normal intervals (SDNN), the root mean square of successive differences (RMSSD), the detrended fluctuations, and sample entropy. In patients in the upright compared to the supine position, SDNN (*p* < 0.001), RMSSD (*p* < 0.001), and total power (*p* = 0.004) all decreased, while the remaining variables were unchanged; no long‐term differences in heart rate variability in the supine position were found between early tilt training and standard care. In the healthy volunteers, all measures besides SDNN and total power changed significantly between supine and upright position. In patients with severe TBI compared to healthy volunteers, several measures of heart rate variability changed differentially during mobilization from the supine to the upright position.

## Graphical abstract

Autonomic regulation of heart rate during mobilization from the supine to the upright position in patients with severe traumatic brain injury. Regulation may be blunted during early mobilization.

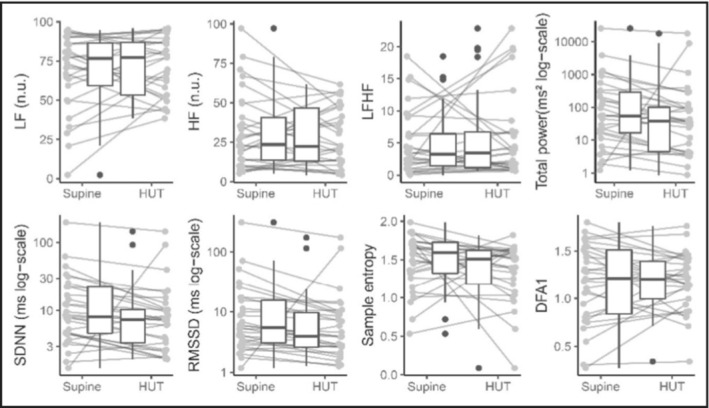

## 1 INTRODUCTION

In the early phase after traumatic brain injury (TBI), autonomic control of cardiovascular function is often compromised (Laver et al., 2014; Lloyd‐Donald et al., 2020; Wijayatilakea et al., 2015), which may contribute to orthostatic intolerance (Baguley et al., 2009; Takahashi et al., 2015) and a need for continuous vasopressor treatment (Lloyd‐Donald et al., 2020), and lead to impaired rehabilitation (Andelic et al., 2012; Laver et al., 2014; Mackay et al., 1992) and potentially worsen long‐term functional outcome.

Orthostatic training by head‐up tilt has been increasingly accepted as an important first step of rehabilitation for patients with a low level of consciousness (Hernandez et al., 2021). In healthy subjects, tilting induces a baroreflex‐regulated autonomic response in order to maintain an appropriate arterial blood pressure and cerebral blood flow (Charkoudian & Rabbitts, 2009; Sato et al., 2017; Stewart, 2012). However, this response may be impaired in patients suffering from TBI, known as orthostatic intolerance; autonomic dysfunction is one potential mechanism for this phenomenon.

Heart rate variability (HRV) is considered a biomarker of sympathetic‐parasympathetic balance within the autonomic nervous system (Malik et al., 1996; Sztajzel, 2004; Taralov et al., 2015; Winchell & Hoyt, 1996). Especially the low‐frequency (LF) to high‐frequency (HF) ratio is believed to represent the activity of the arterial baroreceptor feedback loop (Cygankiewicz & Zareba, 2013). Previously, the LF/HF‐ratio has been found to be lower, both during supine rest and during passive standing on a tilt table in patients with severe acquired brain injury (ABI) compared to healthy controls, indicating reduced baroreceptor activity (Riberholt et al., 2016). Similarly, lower values of various HRV measures have been found in patients with ABI in the sub‐acute rehabilitation phase when compared with healthy controls (Vistisen et al., 2014).

The mechanisms behind orthostatic intolerance and autonomic dysfunction in these patients are incompletely understood. Besides the effects of brain injury per se and sedation, immobilization may also play a role as seen in healthy persons confined to bed rest (Barbic et al., 2019). Thus, general recommendations for other patients with orthostatic hypotension include the avoidance of deconditioning by lower‐limb exercise (Benarroch, 2008; Wieling et al., 2022). Thus, early mobilization and physical activity could potentially improve autonomic balance and orthostatic tolerance after severe TBI, as neural changes and changes in the metabolic system are activity‐dependent (Saltin et al., 1968; Stenger et al., 2012).

This study aimed to examine autonomic control of cardiovascular function and the response to head‐up tilt in patients with TBI, analyzed by position‐dependent changes in HRV. We hypothesized that in the early phase after TBI, (1) the short‐term HRV response to head‐up tilt is impaired, and (2) orthostatic training by head‐up tilt improves long‐term HRV.

## 2 MATERIALS AND METHODS

### 2.1 Study design

The study data was gathered as part of a previously reported randomized feasibility trial investigating the feasibility and safety of early orthostatic exercises in patients with severe TBI (patients) (Riberholt, Olsen, Søndergaard, et al., 2021), and an intra‐observer reliability study (healthy volunteers) (Riberholt, Olsen, Skovgaard, et al., 2021). These studies were conducted in accordance with the latest version of the Helsinki Declaration (World Medical Association, 2013) and the ICMJE Recommendations for the Protection of Research Participants (www.icmje.org). The study protocol has been published (Riberholt et al., 2018), approved by the Scientific‐Ethics Committee of the Capital Region in Denmark (H‐16041794 and H‐16042103), and registered at ClinicalTrials.gov (NCT02924649). The reporting of the trial follows the CONSORT statement and flowchart as well as the TIDieR checklist, found in the previous publication (Riberholt, Olsen, Søndergaard, et al., 2021).

### 2.2 Participants

Participants in the trial were included as a convenience sample of patients admitted to the Neurointensive Care Unit, The Neuroscience Centre, Rigshospitalet, with a clinical diagnosis of severe TBI, a Glasgow Coma Score (GCS) <11 during wake‐up call, a suspected persistent disorder of consciousness, and at least 24 h of stable intracranial pressure (<20 mmHg). Median duration from injury to randomization was 12 days. As the patients included were temporarily incompetent, inclusion depended on informed consent from the next of kin. Patients were excluded if they had spinal cord injuries or fractures of the lower extremities that prohibited weight bearing.

A group of 15 healthy volunteers with no known prior disease were also included (Riberholt, Olsen, Skovgaard, et al., 2021).

### 2.3 Randomization and masking

Patients were randomly assigned (1:1) to 4 weeks of early orthostatic exercise (intervention group) or standard care (control group), using a web‐based computer‐generated block‐randomization procedure set up by the Copenhagen Trial Unit (Centre for Clinical Intervention Research, Rigshospitalet). Block sizes were randomly assigned as either four, six or eight patients. We stratified the randomization according to either low or high GCS (low 3–6; high 7–10). Due to the nature of the intervention (head‐up tilt), masking was not possible neither to the clinical staff nor the patients. All baseline assessments were performed prior to randomization.

### 2.4 Intervention and standard care

The early orthostatic exercise consisted of daily (weekdays) head‐up tilt on an ERIGO® tilt‐table (Hocoma) to 70° for 20 min as this was perceived to be feasible when planning the trial. Vital signs and intracranial pressure were monitored during tilting. In case of a critical reduction in either blood pressure (systolic <80 mmHg; diastolic <50 mmHg) or cerebral perfusion pressure (<50 mmHg), or an increase in either intracranial pressure (>25 mmHg) or heart rate (>180 bpm), the participant was moved to 0° until stable before returning to 70° (Riberholt et al., 2018). Time at 0° was not considered part of the 20‐min session. Participants who regained the ability to stand up during the 4‐week intervention period did not undergo further orthostatic exercise but remained in the study.

Treatment in the standard care group was decided by the treating physician, nurses, and therapists. Mobilization could be a part of the standard care but occurred at a much smaller scale than in the intervention group. The major focus was respiratory optimization and re‐positioning to prevent pressure ulcers, and tilt‐table mobilization was not a part of standard care. For more detailed information we refer to the original publication (Riberholt, Olsen, Søndergaard, et al., 2021) and the published trial protocol (Riberholt et al., 2018).

### 2.5 Measurements

Heart rate variability during head‐up tilt was assessed during a single head‐up tilt session, which was performed before randomization. Two types of electrocardiography (ECG) devices were used for short‐ and long‐term recordings. Short‐term measurements of 5 min were done in supine position and during head‐up tilt at the baseline timepoint using a three‐lead ECG and a bio‐amplifier (FE132 Bio Amp, ADInstruments) at a sample rate of 1 kHz. The data from the device was stored using Labchart (Labchart ver. 8.0, ADInstruments). This allowed us to obtain data that were synchronized with the head‐up tilt procedure. Recordings that were shorter than 5 min because of complications or interference were later excluded from analysis.

Additionally, long‐term recordings were performed on all patients with a device for measuring two‐lead ECG (ePatch‐Biotelemetry, Inc.) at baseline before randomization. The ePatch was placed with an adhesive electrode patch ventrally of the sternum before the first head‐up tilt and recorded continuous ECG for up to 5 days (120 h) at a sample rate of 256 Hz.

In the patient group, the head‐up tilt was performed before randomization and before any of the trial interventions.

Although we did collect arterial blood samples before and during head‐up tilt, we did not measure plasma catecholamines.

### 2.6 HRV analysis of data

The stored data were extracted to and analyzed in Kubios HRV Premium version 3.4.3 (Kubios Oy). All recordings were pre‐processed to remove artifacts. The software uses an *automatic beat detection* algorithm, which filters the ECG signal using a time series consisting of differences between successive RR intervals to identify any ectopic beats or noisy segments (Lipponen & Tarvainen, 2019). Additionally, the 5‐min recordings were visually inspected for any artifacts or arrythmias not caught by the algorithm. Spurious peaks were removed from the analysis and recordings with more than 5% artifacts were excluded from the analysis.

An explorative approach to HRV‐based assessments of autonomic function was pursued, as there is currently no established gold standard (Shaffer & Ginsberg, 2017). Both time domain, frequency domain, and nonlinear metric‐based HRV‐indices are reported (Table 1).

**TABLE 1:** Baseline characteristics.

	All patients (*n* = 30)	Early orthostatic exercise (*n* = 14)	Standard care (*n* = 16)	Healthy volunteers (*n* = 15)
Age (years)—median (IQR)	38 (25–53)	45 (25–53)	37 (25–55)	28 (23–29)
Male—*n* (%)	24 (80%)	11 (79%)	13 (81%)	5 (33%)
First measured GCS—median (IQR)	6 (3–9)	6 (3–9)	5 (3–9)	‐
Sedated at randomization—*n* (%)	9 (30%)	4 (29%)	5 (31%)	‐
RASS—median (IQR)	−3 (−4 to −3)	−3 (−3 to −3)	−4 (−5 to −3)	‐
Days from injury to first head‐up tilt—median (IQR)	12 (10–15)	15 (12–16)	11 (8–13)	‐

Abbreviations: GCS, Glasgow Coma Scale; IQR, interquartile range; RASS, Richmond Agitation and Sedation Scale.

The time domain measures comprised the standard deviation (SD) of successive RR intervals (SD of normal‐to‐normal intervals [SDNN]) and root mean square of successive RR intervals differences (RMSSD). These measures principally reflect the degree of respiratory sinus arrhythmia, which is regulated by parasympathetic activity.

Frequency domain calculations were based on the fast Fourier transform with a 50% Welch window overlap and included total power, LF power (LF: 0.04–0.15 Hz), HF power (HF: 0.15–0.40 Hz) and the ratio between LF and HF power (LF/HF). Total power expresses the signal energy and can be used after normalization to compare the frequency domain between different patients or even between patients and healthy, age‐matched individuals. LF depends mainly on sympathetic, baroreceptor drive, but also on parasympathetic regulation, and to a lesser extent on unspecific factors. HF reflects mainly parasympathetically‐mediated respiratory sinus arrhythmia, while LF/HF is classically considered an index of “sympathetic‐vagal balance” of heart rate regulation, particularly during long‐term recordings.

The nonlinear analysis consisted of sample entropy as a quantification of total irregularity and detrended fluctuations analysis (DFA1). This analysis results in slope α1 and α2, which describes long‐term fluctuations, of which α1 reflects the complex interactions between various heart rate control mechanisms in the arterial baroreflex, while α2 reflects the complex interactions between the regulatory mechanisms that limit fluctuations in heart rate.

### 2.7 Statistical analysis

Statistical analysis was done independently by two investigators (CR and MHO) using SAS/STAT software (SAS Institute Inc.) and R (version 4.1.0, R Core Team), respectively. The plan for statistical analysis was pre‐published at Zenodo.org, and later supplemented by the final statistical report (Olsen & Riberholt, 2022).

The mean and SD were used if variables were normally distributed: if not, data were presented as the median and interquartile range (IQR). A quantile‐quantile plot and the Shapiro–Wilk test were used to investigate distribution. Dichotomous demographic data were presented as numbers and percentages.

To correct for multiple comparisons, we adjusted the alpha level as suggested by Jakobsen et al. (2014), that is, by dividing the original alpha‐level with the midpoint between the level of no correction, equal to one, and the Bonferroni correction, in this case for eight variables; a *p*‐value below 0.01 was thus accepted as significant. If the two independent analyses differed due to software‐specific variations, the most conservative *p*‐value was chosen.

Comparison of HRV in the supine and upright positions and between patients and healthy volunteers in supine was done using paired *t*‐test, or Wilcoxon signed rank test as the equivalent non‐parametric test. Long‐term recordings were analyzed using a mixed‐effects model, with intervention group and time as fixed effects and participants as random‐effect. Both groups received similar interventions on Day 1, while they differed from Day 2 and onwards, and all patients were thus coded as control group on Day 1, and according to their intervention group from Day 2. The mixed effect model was used to investigate possible group‐specific differences over time.

As further post hoc exploratory analyses, we correlated individual HRV measures with each other, with age and days from injury using Pearson correlations coefficient. Finally, we also carried out mixed effects linear regression models investigating if age (corrected for group allocation) and/or mobilization would influence the HRV measures.

## 3 RESULTS

### 3.1 Population

**FIGURE 1:**
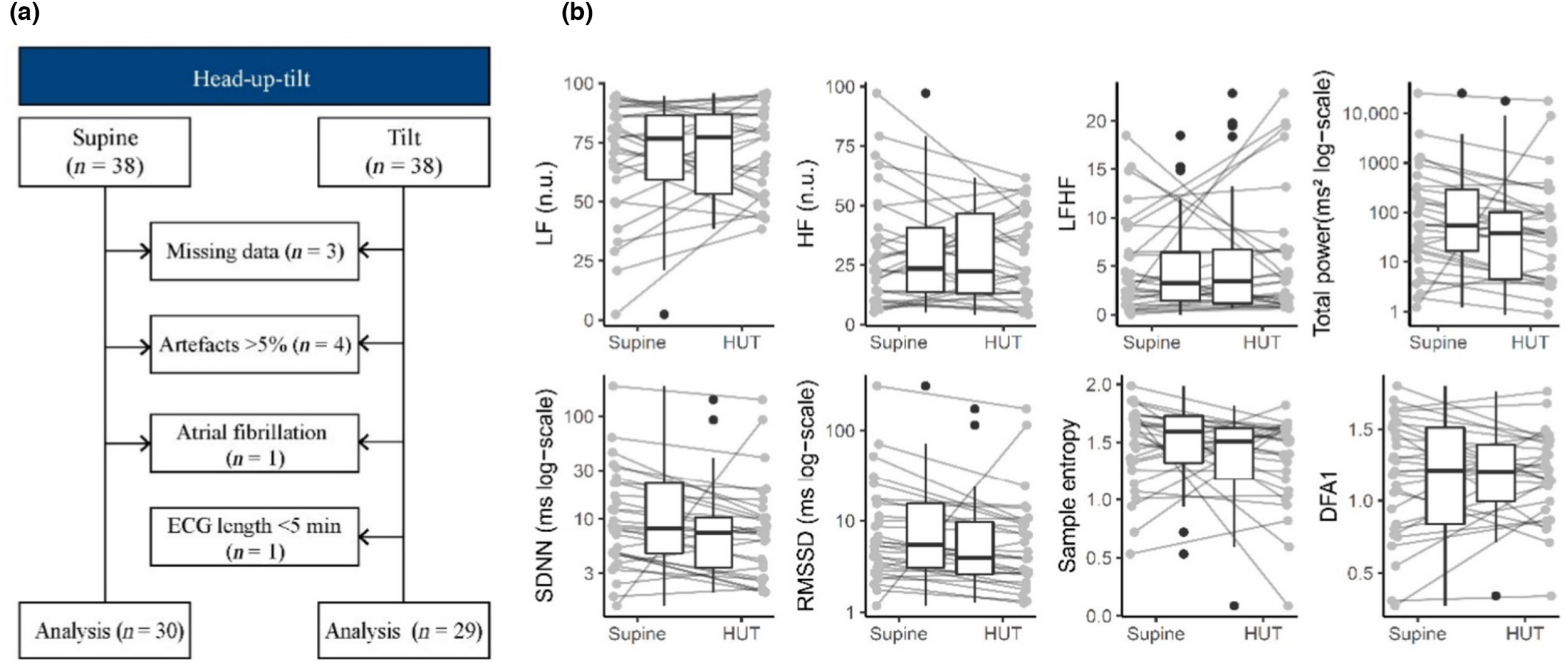
Short‐term heart rate variability (HRV) during head‐up tilt in patients with traumatic brain injury. (a) Flow‐chart of short‐term measurements. (b) HRV analysis of short‐term recordings from head‐up tilt, presented as the median with interquartile range with total power, SDNN, and RMSSD on a log‐scale for better visual presentation. There was a significant difference in total power (*p* = 0.004), SDNN (*p* < 0.001) and RMSSD (*p* < 0.001). To improve the visualization of total power, SDNN and RMSSD the graph is illustrated using a logarithmic scale. For the original scale see Figure S1. DFA1, detrended fluctuation analysis; ECG, electrocardiogram; HF, high‐frequency power (in normalized units [n.u.]); HUT, head‐up tilt; LF, low‐frequency power (in normalized units); LFHF, ratio of LF over HF; RMSSD, root mean square of successive differences; SDNN, standard deviation of normal‐to‐normal beat intervals.

Of the full population of 38 patients, 30 were eligible for final data analysis (Figure 1a). Demographics mirrored the population of the original randomized clinical trial (Table 1). Patients were included on a median of 12 (IQR 10–15) days post ictus and subsequently randomized into equal‐sized groups of intervention and standard care (Table 1). The 15 healthy volunteers were younger than the patient group and with a larger proportion of females (Table 1). The healthy volunteers completed two head‐up tilt sessions approximately 1 month apart, which was identical to the head‐up tilt session performed on patients in the trial. Fourteen volunteers completed two sessions and one only completed the first. Data from both sessions are included in the HRV analysis.

### 3.2 HRV short‐term data

In the patients, five short‐term HRV recordings that did not make it through the pre‐processing procedure were excluded from further analysis. Additionally, one patient suffered from atrial fibrillation, rendering HRV analysis impossible. In healthy volunteers, two recordings (one supine and one during head‐up tilt) did not make it through the pre‐processing procedure and were excluded from further analysis. Thus, data from 30 patients and 15 volunteers were included in the analysis.

**FIGURE 2:**
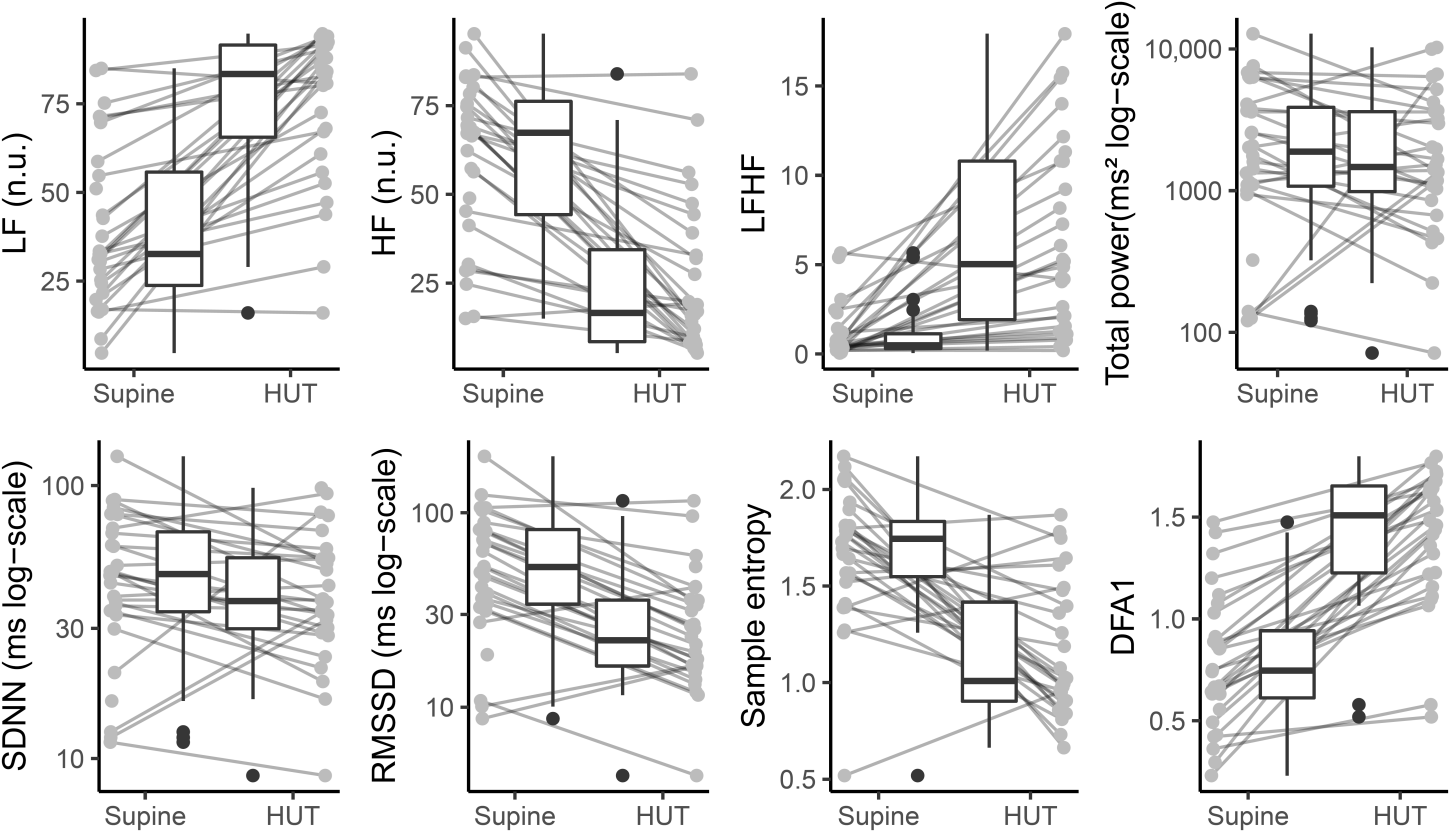
Heart rate variability (HRV) analysis of short‐term ECG between supine and head‐up tilt in healthy volunteers. HRV analysis of short‐term recordings from head‐up tilt presented as the median with interquartile range with total power, SDNN, and RMSSD on a log‐scale for better visual presentation. There was a significant difference in LF (*p* < 0.001), HF (*p* < 0.001), LF/HF (*p* < 0.001), RMSSD (*p* < 0.001), sample entropy (*p* < 0.001), and DFA1 (*p* < 0.001). DFA1, detrended fluctuation analysis; ECG, electrocardiogram; HF, high‐frequency power (normalized units [n.u.]); HUT, head‐up tilt; LF, low‐frequency power (in normalized units); LFHF, ratio of LF over HF; RMSSD, root mean square of successive differences; SDNN, standard deviation of normal‐to‐normal beat intervals.

The HRV measures in the supine position differed between the patient group and the healthy volunteers for all variables except for sample entropy; thus LF, LFHF, and DFA1 were lower, while HF, total power, SDNN, and RMSSD were higher in healthy volunteers compared to patients (Figures 1b and 2b).

During head‐up tilt compared to the supine position in patients, three out of eight HRV measures (SDNN, RMSSD, and total power of the frequency domain) decreased (Figure 1b). By contrast, LF, HF, LF/HF, sample entropy, and DFA1 all remained unchanged. In an exploratory analysis, these results were unchanged after removing extreme outliers.

During head‐up tilt compared to the supine position in the healthy volunteers, three out of eight measures (LF, LF/HF, and DFA1) increased, while HF, RMSSD, and sample entropy decreased tilt (Figure 2). The point estimates of SDNN and total power also decreased, but this was only nearly significant (*p* = 0.02 and *p* = 0.09, respectively).

An exploratory correlation analysis on short‐term HRV measures in the patients suggested an association between age and sample entropy during tilt (*r* = 0.47; *p* = 0.0097). None of the other HRV variables in supine or during head‐up tilt were correlated with age or days from injury. We found significant correlations between HRV measures except for sample entropy and LFHF ratio (Figure S2).

### 3.3 HRV data long‐term

**FIGURE 3:**
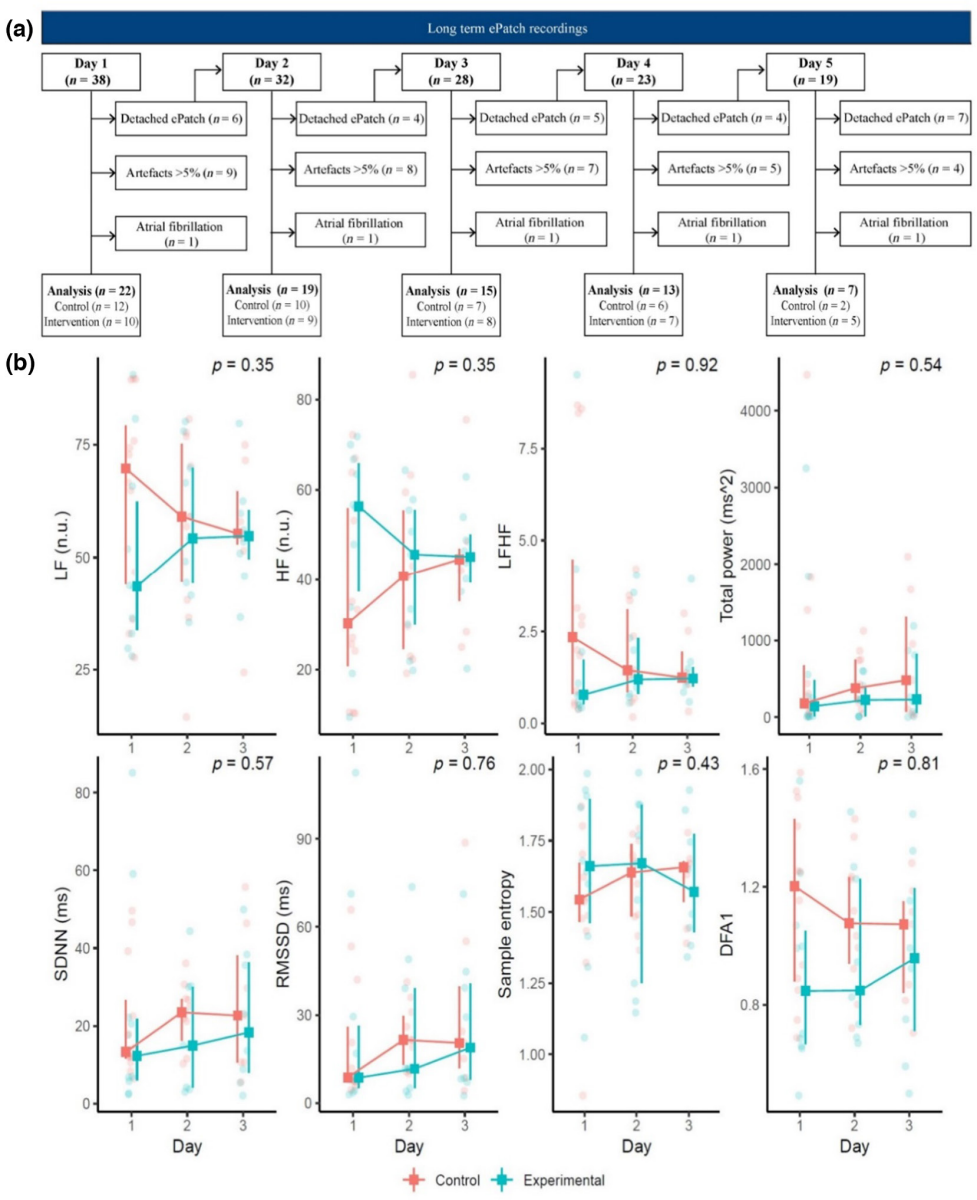
Long‐term ePatch recordings in patients with traumatic brain injury. (a) Flow chart of long‐term measurements. (b) Heart rate variability analysis of long‐term recordings from head‐up tilt, presented as the median with interquartile range for each day. DFA1, detrended fluctuation analysis; HF, high‐frequency power (in normalized units); LF, low‐frequency power (in normalized units); LFHF, ratio of LF over HF; RMSSD, root mean square of successive differences; SDNN, standard deviation of normal‐to‐normal beat intervals.

In several patients, the ePatch was detached before the scheduled 5‐day recording was completed. The reasons for detachment included a need for surgery, CT scanning of the chest, and transfer to other hospitals. Furthermore, recordings from one patient with atrial fibrillation and from patients that did not meet the quality criteria were excluded from analysis. By the end of Days 1, 2, and 3, valid recordings from 22, 19, and 15 patients, respectively, were available for analysis. As 40% or more of data were missing on Days 4 and 5, we decided to restrict the analysis to the first 3 days (Figure 3a).

No difference was found between the control group and the group receiving early tilt training (Figure 3b). HRV variables remained unchanged over time during the 3 days of long‐term recording.

These results were unchanged after the removal of extreme outliers in an exploratory analysis. We also analyzed the effect of group allocation, age, and dose of mobilization on individual HRV variables. This was done in an exploratory linear mixed effects model including group, day, and age as independent variables. In another analysis, we investigated the number of mobilizations performed during the first 3 days and corrected for days. None of the individual HRV variables were significantly affected (Table S2).

## 4 DISCUSSION

This study investigated the autonomic response to tilting in the acute phase of TBI along with potential changes induced by early tilt training. During head‐up tilt in patients, the total power of the frequency domain, SDNN, and RMSSD all decreased, whereas the remaining HRV measures remained unchanged. This was contrary to the reference group of healthy volunteers as HF and sample entropy were lower during head‐up tilt and LF, LF/HF, and DFA1 increased from supine to head‐up tilt. Further, the comparison at baseline revealed that the patients in the supine position had lower values of HF, total power, SDNN, and RMSSD while LF, LFHF, and DFA1 slopes were higher when compared to the healthy volunteers. As an additional finding, no change in the long‐term HRV analyses was found between patients receiving early tilt training compared to standard care within the first 3 days of intervention.

The primary aim of early enrolment and tilting of TBI patients was achieved with a mean inclusion point of 12.8 days post‐event and a tilt to 70° of all patients with short‐term recordings. However, the long‐term recordings proved to be challenging within the context of intensive care. In order not to diminish sample size further, analyses of long‐term recordings were restricted to the first 3 days of recording. This meant a very short period to observe possible effects of the intervention. It would have been interesting to do a follow‐up of heart rate from the rehabilitation phase, however, this was not the primary focus of the study.

To the best of our knowledge, this is the first study to report on the autonomic response to an orthostatic challenge in the acute phase after TBI. The observed tilt‐induced reductions in SDNN, RMSDD, and total power in short‐term recordings corresponded qualitatively to those of the included group of healthy volunteers and those which have been previously reported in healthy individuals (Montano et al., 1994; Pawłowski et al., 2021; Piccirillo et al., 1995; Sharma et al., 2009; Stewart, 2000). The findings probably reflect increased sympathetic drive before and during the head‐up tilt. A previous study of patients with TBI in the sub‐acute rehabilitation phase found no response in HRV and heart rate to activities such as walking, standing, and sitting (Katz‐Leurer et al., 2014). We found no change in LF, HF, or the LF/HF ratio between supine to the standing position in the short‐term recordings, and no general changes in the long‐term recordings in the patients. The LF and HF have been shown to correlate with increasing tilt‐table angle in healthy persons, and we would expect to see an increase in the LF during tilt (Montano et al., 1994) and a decrease in the HF resulting in a higher LFHF ratio. This would also concur with the observed reductions in SDNN, RMSDD, and total power seen in both patients and healthy volunteers. However, our findings are in line with previous data showing no difference in the supine position during the first days after TBI (Hendén et al., 2014) or in the later phase during head‐up tilt (Riberholt et al., 2016). The lack of increase in particularly the LF domain could indicate reduced arterial baroreceptor sensitivity, which in part could be due to the sedation or general medication given at the intensive care unit, immobilization, and the extent of the brain injury. The missing change in the DFA1 in the long‐term recordings on Day 3 may corroborate this notion. Alternatively, a high sympathetic drive in the supine position (as indicated in the comparison between patients and healthy volunteers) could have induced a “ceiling effect,” so that no further sympathetic activation was possible during the head‐up tilt. Although not statistically significant, the DFA1 trending towards 1.05 in the long‐term measurements on Day 3 could indicate some normalization of arterial baroreflex function in both groups (Kleiger et al., 2005). Both the short‐ and long‐term sample entropy recordings support the lack of change seen in the patients, as this expresses the regularity and complexity of the recorded time series (Henriques et al., 2020). Future studies are needed to confirm these results, but studies examining cardiovascular regulation or early mobilization should be concerned with these signs indicating reduced cardiovascular regulation.

The primary strength of this study is that the design included an autonomic challenge in the acute phase; this has not previously been attempted so early after TBI. The major limitations were the small sample size and the large amount of missing data. In both the long‐term and short‐term analysis some extreme outliers were present (Figures 1b and 3b). When we removed these, as a post hoc exploratory analysis, the results did not change. One‐third of the patients were sedated during tilting, which could have driven the results in a less responsive direction as it is unknown to what degree sedation may affect the autonomic cardiac response to an orthostatic challenge. Depending on sedation level and settings of the mechanical ventilator, the respiratory frequency will have some impact on the HF band. This, in comparison, is not the case with the healthy volunteers. HRV requires stable recordings of ECG and normal sinus rhythm, no atrial fibrillation, sinoatrial dysfunction, and a low number of ectopic complexes (Kleiger et al., 2005; Voss et al., 2009). This may result in a selection bias in the data from the trial. Finally, this was an exploratory study in the clinical setting; the population varied in demography, injury severity, sedation level and comorbidities, all of which may affect physiological state and HRV response.

## 5 CONCLUSIONS

Our findings suggest that patients in the early phase after TBI exhibit a blunted cardiac autonomic response to tilting according to several HRV metrics. We speculate that an increased sympathetic activity in the supine position in patients, which could not be further increased during head‐up tilt, may explain this blunted response. However, this hypothesis as well as the role of methodological limitations and sedation remains to be further investigated. This study extends earlier research by investigating the autonomic response to tilting in the acute phase, rather than later during rehabilitation. Patients may benefit from an increased focus on possible impairment of the autonomic regulation, including orthostatic intolerance, to improve early rehabilitation.

## AUTHOR CONTRIBUTIONS

Conceptualisation and design: MS, RMGB, JM, KM, and CGR; Acquisition: CGR and MS; Data validation, MS, MA, MHO, and CGR; statistical analysis, MHO and CGR; writing—original draft preparation, MS; critical revising, MS, MA, MHO, RMGB, JM, KM, and CGR; funding acquisition, CGR. All authors qualify for authorship, have read and approved the final version of the manuscript, agree to be accountable for authorship, and all those who qualify for authorship are listed.

## FUNDING INFORMATION

This study was funded by The Council of Danish Victims Fund (grant 16‐910‐00043), by the Research Fund of Rigshospitalet, Copenhagen University Hospital (R114‐A4672), and the Danish Physical Therapy Association (15242) through grants to CGR.

## CONFLICT OF INTEREST STATEMENT

The authors declare no conflict of interest. The funders had no role in the design, analyses, interpretation of data, writing of the manuscript, or in the decision to publish the results.

## ETHICS STATEMENT

The study was conducted in accordance with the Declaration of Helsinki and approved by The Regional Ethics Committee of the Capital Region in Denmark (H‐16041794).

## INFORMED CONSENT STATEMENT

Informed consent was obtained from next of kin to all subjects involved in the study. Healthy volunteers all gave informed consent.

## Supporting information


Data S1.
