# Supplementary material for: Autonomic response to early head‐up tilt in patients with severe traumatic brain injury: Analysis from a randomized feasibility trial
Source: Physiol Rep. 2023 Apr 20;11(8):e15666. doi: 10.14814/phy2.15666 (PMC10116542; doi:10.14814/phy2.15666)
Supplement: Supplementary file 1 — Data S1. [file PHY2-11-e15666-s001.docx]

Supplemental material

Table S1. HRV indices and interpretation

| **Variable** | **Definition** | **Interpretation** |
| --- | --- | --- |
| SDNN (ms) | Standard deviation of normal-to-normal beat intervals. | Reflects all components of the autonomic regulation. Measure of total variability.* [14–16, 33–35] |
| RMSSD (ms) | Root mean square of successive differences, between normal-to-normal beat intervals. | Measure of short-term, beat-to-beat, variability. Reflects the parasympathetic regulation of heart rate. [14–16, 33–35] |
| LF (n.u.) | Low-frequency power; 0.04-0.15Hz | Reflects both parasympathetic and sympathetic activity, though correlated to mental and physical stress – e.g. physical activity, bleeding, tilting, coronary occlusion  Presented in normalised units. representing the relative value of the component in proportion to the total power.  [14–16, 33–35] |
| HF (n.u.) | High-frequency power; 0.15-0.4Hz | Reflects parasympathetic activity; correlated to respiratory sinus arrhythmia.  Presented in normalised units. representing the relative value of the component in proportion to the total power.  [14–16, 33–35] |
| LF/HF | The ratio of LF over HF | Balance between the sympathetic and parasympathetic regulation. [14–16, 33–35] |
| Total Power (ms^2^) | All frequencies | Reflects all components of the autonomic regulation. Measure of total variability.* [14–16, 33–35] |
| Sample entropy | The negative natural logarithm of the conditional probability that a dataset of length *N*, having repeated itself within a tolerance of *r* for *m* points, will also repeat itself for *m+1* points, not allowing self-matches. | Overall complexity and predictability of HRV in the time-series.* [14, 39, 40] |
| DFA1 | Detrended fluctuation analysis | A measure of the degree to which the RR interval pattern is random at one extreme or correlated at the other [39, 40]. |

*The autonomic regulation of heart rate is the result of complex interactions from different circulatory components – e.g., baro- and chemoreceptors, sympatho-sympathetic reflexes, hormonal factors, and central oscillations. All of these contribute to the overall variability of heart rate. A dominance of one factor due to a physiological rise or pathological diminished signals from others will reduce the complexity of the HRV. n.u.: normalised units.

**Table S2. Linear mixed effects model of heart rate variability variables and the influence of age or mobility.**

|  | **A.** | **Value** | **SE** | **p-value** |  | **B.** | **Value** | **SE** | **p-value** |
| --- | --- | --- | --- | --- | --- | --- | --- | --- | --- |
| **LF** | *Group* | -9.18 | 7.41 | 0.2277 |  |  |  |  |  |
|  | *Day* | 1.29 | 2.34 | 0.5847 |  | *Day* | 1.31 | 2.34 | 0.5790 |
|  | *Age* | 0.06 | 0.22 | 0.8045 |  | *Mob* | -4.30 | 2.40 | 0.0853 |
| **HF** | *Group* | 9.09 | 7.39 | 0.2313 |  |  |  |  |  |
|  | *Day* | -1.30 | 2.33 | 0.5822 |  | *Day* | -1.32 | 2.33 | 0.5761 |
|  | *Age* | -0.06 | 0.22 | 0.7933 |  | *Mob* | 4.27 | 2.39 | 0.0866 |
| **LFHF** | *Group* | -1.25 | 0.89 | 0.1706 |  |  |  |  |  |
|  | *Day* | -0.32 | 0.26 | 0.2292 |  | *Day* | -0.33 | 0.26 | 0.2263 |
|  | *Age* | 0.01 | 0.03 | 0.7342 |  | *Mob* | -0.47 | 0.29 | 0.1224 |
| **totP** | *Group* | -69.78 | 257.64 | 0.7889 |  |  |  |  |  |
|  | *Day* | -102.07 | 128.88 | 0.4348 |  | *Day* | -126.33 | 129.13 | 0.3360 |
|  | *Age* | -11.50 | 7.60 | 0.1441 |  | *Mob* | 21.42 | 92.58 | 0.8190 |
| **SDNN** | *Group* | 4.04 | 5.98 | 0.5067 |  |  |  |  |  |
|  | *Day* | -0.94 | 2.20 | 0.6714 |  | *Day* | -1.32 | 2.19 | 0.5510 |
|  | *Age* | -0.34 | 0.18 | 0.0687 |  | *Mob* | 1.93 | 2.15 | 0.3788 |
| **RMSSD** | *Group* | 8.43 | 8.69 | 0.3421 |  |  |  |  |  |
|  | *Day* | 0.01 | 3.32 | 0.9969 |  | *Day* | -0.36 | 3.30 | 0.9149 |
|  | *Age* | -0.34 | 0.26 | 0.1997 |  | *Mob* | 4.18 | 2.95 | 0.1702 |
| **Entropy** | *Group* | 0.01 | 0.09 | 0.9275 |  |  |  |  |  |
|  | *Day* | 0.03 | 0.03 | 0.2576 |  | *Day* | 0.04 | 0.03 | 0.2272 |
|  | *Age* | 0.00 | 0.00 | 0.1707 |  | *Mob* | 0.00 | 0.03 | 0.9905 |
| **DFA1** | *Group* | -0.18 | 0.11 | 0.1282 |  |  |  |  |  |
|  | *Day* | 0.01 | 0.03 | 0.7111 |  | *Day* | 0.01 | 0.03 | 0.6826 |
|  | *Age* | -0.00 | 0.00 | 0.1584 |  | *Mob* | -0.08 | 0.04 | 0.0436 |

Linear mixed effects model of heart rate variables using participants as a random effect and *either* A. group (Early orthostatic exercise or standard care), day (1 to 3), and age or B. day (1 to 3) and number of times mobilised during the first three days, as fixed effects. DFA1: Detrended fluctuation analysis; HF: High-frequency power (in normalised units); LF: Low-frequency power (in normalised units); LFHF: Ratio of LF over HF; Mob: Number of mobilisations within the first three days; SDNN: Standard deviation of normal to normal beat intervals; SE: Standard error; RMSSD: Root mean square of successive differences.

**Figure S1. HRV analysis of short-term analysis between supine and HUT in patients with traumatic brain injury.**


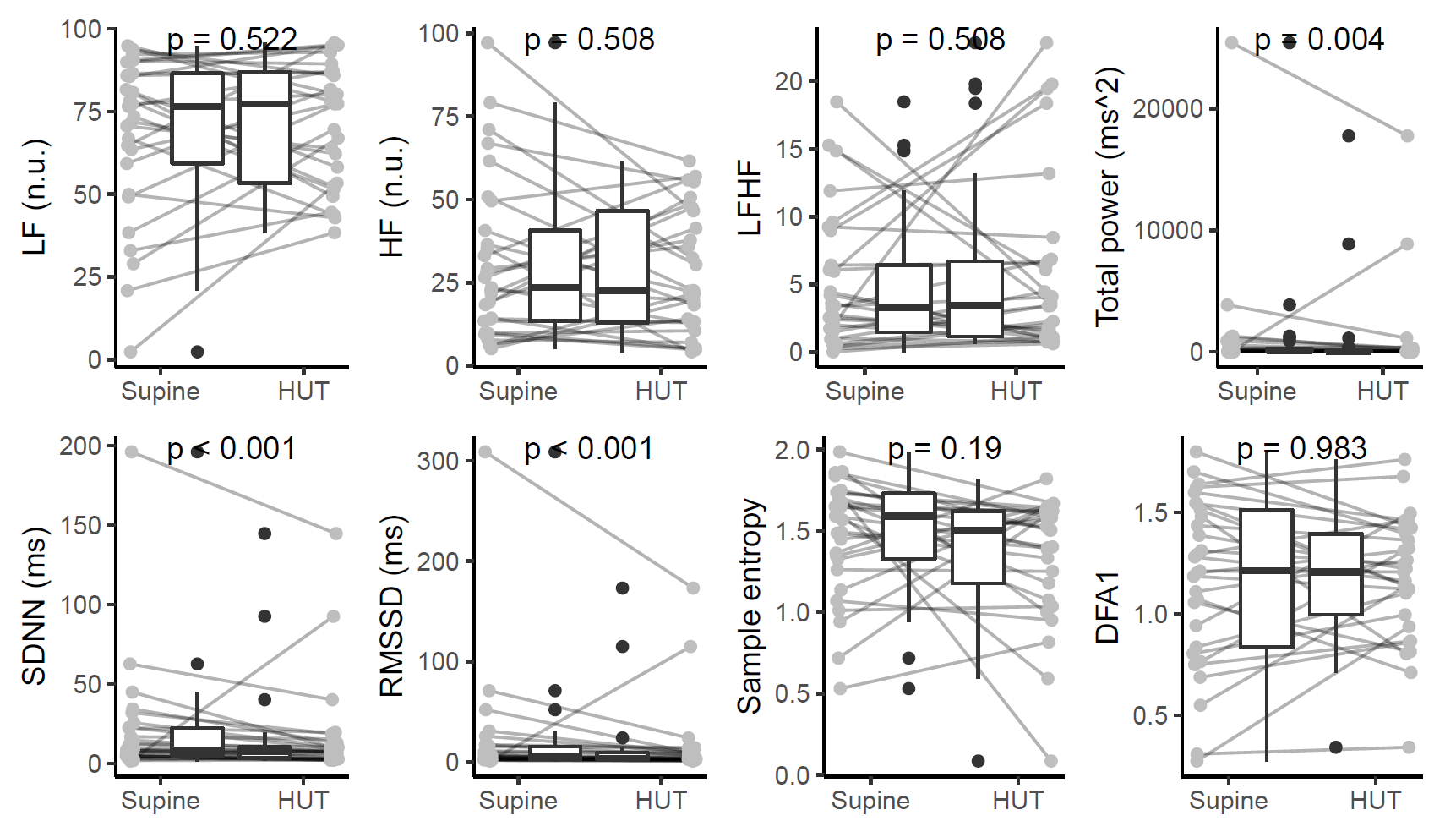


HRV analysis of short-term recordings from head-up tilt presented as the median with interquartile range. DFA1: Detrended fluctuation analysis; HF: High-frequency power (in normalised units); HUT: Head-up tilt; LF: Low-frequency power (in normalised units); LFHF: Ratio of LF over HF; SDNN: Standard deviation of normal to normal beat intervals; RMSSD: Root mean square of successive differences


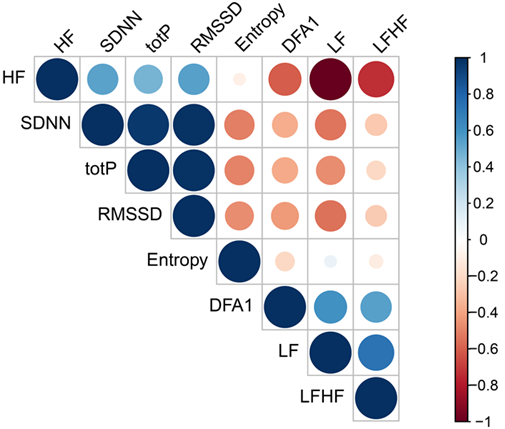
Figure S2. Correlation between HRV variables in supine position

|  |  | LF | HF | LFHF | totP | SDNN | RMSSD | Entropy |
| --- | --- | --- | --- | --- | --- | --- | --- | --- |
| HF | R  P-value | -1  ***<.0001*** |  |  |  |  |  |  |
| LFHF | R  P-value | .7378  ***<.0001*** | -.7377  ***<.0001*** |  |  |  |  |  |
| totP | R  P-value | -.4682  ***.0002*** | .4669  ***.0002*** | -.2030  *.1231* |  |  |  |  |
| SDNN | R  P-value | -.5393  ***<.0001*** | .5385  ***<.0001*** | -.2663  *.0415* | .9679  ***<.0001*** |  |  |  |
| RMSSD | R  P-value | -.5475  ***<.0001*** | .5463  ***<.0001*** | -.2544  *.0519* | .9848  ***<.0001*** | .9819  ***<.0001*** |  |  |
| Entropy | R  P-value | .0807  *.5434* | -.0801  *.5466* | -.1046  *.4305* | -.4907  ***<.0001*** | -.5076  ***<.0001*** | -.4615  ***.0002*** |  |
| DFA1 | R  P-value | .6095  ***<.0001*** | -.6076  ***<.0001*** | .5455  ***<.0001*** | -.3786  ***.0031*** | -.3666  ***.0043*** | -.4282  ***.0007*** | -.2060  *0.1176* |

Pearson correlation coefficient between the different HRV variables. The figure to the left presents the correlations graphically with the colour indicating the correlation and the size of the circles indicates the *P*-value (larger circles equals lower *P*-values). All correlation coefficients were significant, except for those between entropy and HF, LF, LFHF, and DFA1 and between LFHF and totP. HF: High-frequency power (in normalised units); SDNN: Standard deviation of normal-to-normal beat intervals; totP: total power; RMSSD: Root mean square of successive differences; DFA1: Detrended fluctuation analysis; LF: Low-frequency power (in normalised units); LFHF: Ratio of LF over HF.
